# Empagliflozin attenuates dexamethasone-induced non-alcoholic steatohepatitis by regulation of ferroptosis, inflammation and autophagy

**DOI:** 10.3389/fphar.2026.1741926

**Published:** 2026-03-18

**Authors:** Naif S. Alharbi, Marwa E. Abdelmageed, Manar A. Nader, Marwa S. Serrya

**Affiliations:** 1 Department of Pharmacology and Toxicology, Faculty of Pharmacy, Mansoura University, Mansoura, Egypt; 2 Department of Pharmacology and Toxicology, Faculty of Pharmacy, Mansoura National University, Gamasa, Egypt

**Keywords:** autophagy, dexamethasone, empagliflozin, ferroptosis, inflammation, non-alcoholic steatohepatitis

## Abstract

**Purpose:**

Non-alcoholic steatohepatitis (NASH) is characterized by hepatic steatosis, inflammation, fibrosis and offers potential for the development of cirrhosis or hepatocellular carcinoma if left untreated. This study was designed to examine the potential preventive effect of the SGLT2 inhibitor empagliflozin (EMPA) against Dexamethasone (DEXA)-induced NASH in *Wistar* rats.

**Methods:**

NASH was induced by daily injection of DEXA (8 mg/kg/day, i.p.) from day 8–13. EMPA (10 and 30 mg/kg) was administered orally for 13 days from day 1–13. on day 14, serum and liver tissue were obtained and assessed using biochemical and histological assessments.

**Results:**

EMPA (10 and 30 mg/kg) considerably ameliorated NASH brought on by DEXA. A marked decrease in serum levels of ALT and AST was observed upon EMPA treatment confirmed by histopathological analysis. EMPA significantly improved metabolic parameters, as evidenced by reductions in serum glucose and insulin levels and a marked improvement in HOMA-IR. EMPA significantly ameliorated dyslipidemia by reducing serum levels of TG, TC, LDL-C, VLDL-C, and FFA, while partially restoring serum HDL-C levels. Hepatic oxidative stress markers, MDA and 4-HNE were markedly reduced, whereas hepatic antioxidant defences, Nrf2, GSH and GPX4 were significantly enhanced. Furthermore, EMPA effectively restored iron homeostasis and alleviated signs of iron overload by down-regulating serum iron and upregulation of hepatic ferritin, transferrin, and hepcidin. EMPA treatment led to a marked decrease in hepatic calcium and calcineurin A levels, which was associated with modulation of autophagy, as evidenced by increased LC3-II and decreased p62 and Beclin-1 expression in the liver. EMPA downregulated pro-inflammatory and fibrotic biomarkers, NF-κB, IL-6, and TGF-β1, as well as markers for hepatic lipid accumulation, FABP1, PPAR-γ and CD36. It also significantly suppressed the hepatic gene and protein expression of NCOA4, the ferritinophagy biomarker, with enhanced FTH1 hepatic gene and protein expression.

**Conclusion:**

EMPA effectively ameliorated DEXA-induced NASH via reducing liver damage caused by excess iron by restoring the appropriate levels of iron, preventing ferroptosis, restoring lipid homeostasis, reducing oxidative stress, and managing inflammation and fibrosis.

## 1 Introduction

The most serious type of Non-alcoholic fatty liver disease (NAFLD), which signifies that the liver stores excessive amounts of fat, is known as non-alcoholic steatohepatitis (NASH). NASH is characterized by hepatic steatosis, inflammation, fibrosis, and offers the potential for the development of cirrhosis or hepatocellular carcinoma if left untreated (Younossi and Henry, 2022). NASH prevalence has increased over the last years and mainly affects adult populations with obesity and type 2 diabetes mellitus (T2DM). Currently, anti-NASH treatment at the global level is comparatively scarce, and, therefore, possible therapies are a continuously emerging indication (Staels et al., 2023).

Dexamethasone (DEXA) is a synthetic glucocorticoid widely used throughout clinical practices because of its potent anti-inflammatory and immune modulatory effect. However, when DEXA is used for the long term, adverse metabolic consequences like change in insulin sensitivity, the presence of dyslipidemia, and hepatic steatosis, which resembles the development of NASH, have been depicted in both human and various experimental models (Peng et al., 2020). In human, exposure to DEXA induces systemic insulin resistance, enhanced hepatic lipogenesis, impaired fatty acid oxidation, and pro-inflammatory signaling, leading to hepatic steatosis and liver injury resembling NASH (Polyzos and Targher, 2024; Rahimi et al., 2020). These studies of NASH in a DEXA-treated model show that this compound affects several pathways essential for lipid homeostasis and inflammatory signaling in hepatocytes (Xiang et al., 2022). This led to the emergence of dexamethasone-induced NASH models as the models for pre-clinical evaluation in determining therapeutic targets.

Ferroptosis, an iron dependent cell death, is markedly related to NASH progression (Tsurusaki et al., 2019). Ferroptosis is a regulated form of cell death driven by the excessive accumulation of polyunsaturated phospholipid hydroperoxides and the failure of lipid repair mechanisms (Kagan et al., 2017). The accumulation of lipid peroxides compromises membrane integrity by disturbing ion homeostasis, decreasing membrane fluidity, and enhancing permeability, which eventually results in membrane rupture and cell death (Feng and Stockwell, 2018). This lipid peroxidation can occur through enzymatic pathways involving lipoxygenases and cytochrome P450 oxidoreductase (POR) (Yan et al., 2021; Zou et al., 2020), or via non-enzymatic mechanisms mediated by iron-dependent Fenton reactions (Conrad and Pratt, 2019). In contrast, three major defense systems—glutathione peroxidase 4 (GPX4), apoptosis-inducing factor mitochondria-associated 2 (AIFM2/FSP1), and dihydroorotate dehydrogenase (DHODH)—counteract ferroptosis by reducing lipid peroxides and maintaining membrane stability (Yang et al., 2014; Bersuker et al., 2019; Mao et al., 2021). Recent studies have shown that ferroptosis is closely related to autophagy, especially ferritinophagy and lipophagy, two types of selective autophagy (Li N. et al., 2020; Bai et al., 2019). The activation of autophagy is critical for cell survival and tissue homeostasis; however, the degradation products of autophagy may have adverse effects autophagy (Li N. et al., 2020; Bai et al., 2019).

Multiple metabolic and signaling pathways have been identified as key regulators of ferroptotic sensitivity (Zheng and Conrad, 2020). Iron, through its incorporation into heme and iron–sulfur clusters, forms the catalytic center of numerous metabolic enzymes. While essential for growth, excessive iron enhances oxidative stress by promoting Fenton reactions and lipid peroxidation, though the exact mechanisms remain partially understood. Iron overload is thus a hallmark of ferroptosis, and cellular iron uptake, storage, utilization, and export collectively control the labile iron pool and lipid oxidation (Zheng and Conrad, 2020). The transferrin receptor (TFRC) mediates iron uptake via clathrin-dependent endocytosis, and its knockdown markedly inhibits ferroptosis by reducing intracellular iron (Bogdan et al., 2016; Gao et al., 2015). Cytosolic iron is subsequently used for heme and iron–sulfur cluster biosynthesis. Loss of NFS1, a cysteine desulfurase essential for iron–sulfur cluster assembly, sensitizes cells to ferroptosis, whereas ISCU overexpression attenuates it (Alvarez et al., 2017; Du et al., 2019). Ferritin and mitochondrial ferritin (FTMT) sequester excess iron, which can be released through NCOA4-mediated ferritinophagy. Under iron deficiency, NCOA4 binds ferritin and LC3, directing ferritin to autophagic degradation, and releasing iron to replenish the labile pool; during iron sufficiency, NCOA4 is degraded to maintain basal ferritinophagy (Mancias et al., 2014; Dowdle et al., 2014). Activation of NCOA4-mediated ferritinophagy enhances ferroptosis, while its inhibition lowers labile iron and suppresses cell death (Hou et al., 2016; Gao et al., 2016). Loss of ferritin heavy chain (*F*th1) in cardiomyocytes or neurons increases susceptibility to iron-induced oxidative injury and ferroptosis, effects that can be alleviated by ferrostatin-1 (Fang et al., 2020; Rui et al., 2021). Collectively, these results emphasize the pivotal function of ferritin in regulating intracellular iron levels and preventing excessive lipid peroxidation. Moreover, lipid metabolic processes covering lipid synthesis, storage, transport, and oxidation further define the abundance of peroxidation-prone polyunsaturated fatty acids and thus critically determine ferroptotic vulnerability (Zheng and Conrad, 2020).

Empagliflozin (EMPA) is a sodium-glucose cotransporter-2 (SGLT2) inhibitor that helps manage type 2 diabetes mellitus by increasing the rate at which glucose could be expelled from the body through urine. SGLT2 inhibitors reduce HbA1c, body weight, and insulin resistance, which are factors that contribute to the progression of NASH state (Mirarchi et al., 2022). In addition to this, the drug contributes to glycosuria that in a way aids in the decrease in fatty liver content and significantly impacts hepatic steatosis (Mehtani and Saigal, 2023). EMPA seems to exert specific actions on inflammation and liver fibrosis, which are essential for confronting the most severe aspects of NASH. Experimentations done in animal models have established that with EMPA, oxidative stress is lessened, production of pro-inflammatory cytokines is reduced, and mitochondrial dysfunction is improved, which has a direct effect on the damage and fibrosis experienced by the liver (Perakakis et al., 2021). Other than preventing further accumulation of fibrosis, which is a critical aspect of NASH, EMPA can address other vascular complications like portal hypertension and can fill the gap of a multi-modal treatment regimen for NASH (Lonardo et al., 2024). Through impacting such pathological processes, EMPA may be capable of providing a remedy not only to the metabolic abnormalities’ characteristic of NASH but also to the fibrotic and inflammatory components of this disease.

NASH induced by DEXA and associated lipid homeostasis and inflammatory signaling in hepatocytes alteration is related to iron overload, and consequently ferroptosis and related autophagy, especially ferritinophagy and lipophagy, is not yet illustrated to our knowledge. Thus, in this study, iron overload and related alteration in DEXA induced NASH in rats was studied, as well as possible ameliorative effects of EMPA.

## 2 Materials and methods

### 2.1 Animals and ethical approval

The research adopted 16 adults male *Wistar* rats of body weight of 200–250 g. Rats were kept under a standard laboratory environment at a temperature of 22 °C ± 2 °C, a light and dark cycle of 12:12 h, and under Ad lib feeding. The current experiments followed previously set standard operating protocols established by the International Animal Care and Use Committee guidelines, Mansoura University, and were also endorsed by the MU-ACUC (PHARM.PHD.23.12.32). The animal study was conducted in compliance with ARRIVE guidelines.

### 2.2 Chemicals and reagents

Empagliflozin (EMPA) is under the brand name of Jardiance (developed by the collaboration of Boehringer Ingelheim and Eli Lilly and Company). DEXA was purchased as Dexamethasone sodium phosphate (8 mg/2 mL ampoule) from Amriya Pharmaceutical Industries (Alexandria, Egypt). All other chemicals and reagents used in the current work were of high analytical grade.

### 2.3 Experimental design

NASH was induced by daily injection of DEXA (8 mg/kg/day, i.p.) on days 8–13. EMPA was suspended in carboxymethylcellulose (CMC) and used at doses of 10 and 30 mg/kg. The resource equation used for determination of the sample size of rats used in this study was done according to previous studies (Arifin and Zahiruddin, 2017; Charan and Kantharia, 2013). Rats were randomly divided into four groups as follows:

Control Group (n = 4): rats received 0.5% CMC (2.5 mL/kg/day, orally) for 13 days.

DEXA Group (n = 4): rats received 0.5% CMC (2.5 mL/kg/day, orally) for 13 days along with a daily injection of DEXA (8 mg/kg/day, i.p.) on days 8–13.

EMPA10/DEXA Group (n = 4): Rats received EMPA (10 mg/kg/day, orally) for 13 days along with administration of DEXA (8 mg/kg/day, i.p.) on days 8–13.

EMPA30/DEXA Group (n = 4): Rats received EMPA (30 mg/kg/day, orally) for 13 days along with administration of DEXA (8 mg/kg/day, i.p.) on days 8–13.

The doses of EMPA and DEXA and the duration of treatment were selected based on the results of preliminary studies to reproduce the results and provide relevance for the corresponding therapy (Amer et al., 2024b; Zaghloul et al., 2022), respectively.

On the 14th day the rats were euthanized by secobarbital (50 mg/kg, i.p.) (Amer et al., 2024a). Blood samples via retro-orbital puncture were collected, centrifuged at 3,000 rpm for 10 minutes, and serum was extracted to be used in biochemical analysis. Rats were then sacrificed by cervical displacement.

Hepatic tissues were removed, rinsed with cold phosphate buffered saline (PBS) to remove blood and debris and slows enzymatic degradation, which is essential for maintaining sample integrity during early handling steps. Dividing the tissue into 3 separate parts is routinely recommended to allow proper preservation for different downstream analyses (e.g., histology, biochemical assays, and molecular studies), following evidence-based biospecimen handling guidelines (Ahmed et al., 2022; Amer et al., 2024b) of liver tissues was stored immediately at −80 °C for later homogenization, and the third part was kept in glutaraldehyde to endure transmission electron microscopy (TEM).

### 2.4 Assay of metabolic parameters

Serum levels of fasting blood glucose were determined using Spinreact colorimetric kit (Santa Coloma, Spain, cat no MD41011). Serum levels of fasting insulin are estimated using BioVendor Group ELISA kits (Ashville, North Carolina, United States, cat no RTC018R). Homeostatic Model Assessment of Insulin Resistance (HOMA IR) is calculated according to the following equation: HOMA-IR = (Fasting Serum Insulin (mU/L) × Fasting Serum Glucose (mmol/L))/22.5 (Abdelmageed et al., 2019).

### 2.5 Assay of liver function parameters

Serum concentration of alanine aminotransferase (ALT) and aspartate aminotransferase (AST) was measured using commercially available kits that were obtained from Spinreact (Santa Coloma, Spain, cat no. 1001170 and MD41264, respectively) according to the instructions provided by the manufacturers.

### 2.6 Histopathological examination

The collected liver tissue samples were fixed in 10% neutral-buffer formalin for 24 h before being embedded in paraffin and sectioned at 5 µm using a microtome for histological evaluation using the Hematoxylin and Eosin (H&E) Stain. Images from the created slides were observed and analyzed using a light microscope (Nikon Eclipse E200, Japan) to determine changes in hepatocyte steatosis, inflammation and fibrosis. The severity of steatosis was assessed semi-quantitatively on a scale of 0–3; lobular inflammation on a scale of 0–3; and hepatocyte ballooning was estimated on a scale of 0–2 using NAS- NASH clinical research network scoring system.

### 2.7 Transmission electron microscopy (TEM)

Liver specimens (∼1 mm^3^) were immediately fixed in 2.5% glutaraldehyde in 0.1 M phosphate buffer (pH 7.4) at 4 °C for 24 h after harvesting. Following primary fixation, specimens were rinsed in phosphate buffer and post-fixed in 1% osmium tetroxide for 2 h. Samples were then dehydrated through a graded ethanol series (30%–100%) and embedded in epoxy resin. Ultrathin sections (∼70 nm) were cut using an ultramicrotome and mounted on copper grids. Sections were stained with uranyl acetate and lead citrate to enhance contrast. Electron micrographs were obtained using a transmission electron microscope at an accelerating voltage of 80 kV.

### 2.8 Assay of iron homeostasis biomarkers

Serum iron was quantified using the colorimetric assay kit (Ferrozine method) purchased from AdipGen® Life Sciences (San Diego, United States, cat no. JAI-CFE-005). Hepatic ferritin was estimated using Eagle Biosciences ELISA kit (NH, United States, cat no. FRR31-K01). Hepatic transferrin and hepcidin levels were measured using the Assay Genie ELISA kit (Dublin, Ireland, cat no. RTDL01062 and RTFI00856, respectively). Hepatic levels of ferritin heavy chain (FTH1) were determined using the AssayGenie ELISA kit (Dublin, Ireland, cat no. RTEB0314). In addition, Hepatic gene expression of FTH1 was estimated by PCR as mentioned in Section 2.15.

### 2.9 Assay of oxidative stress-related biomarkers

Hepatic contents of malondialdehyde (MDA) and 4-hydroxynonenal (4HNE) were estimated using commercially available ELISA kits that were obtained from MyBioSource (California, United States, cat no. MBS268427) and Assay Genie (Dublin, Ireland, cat no. RTF101293), respectively following the instructions of the manufacturers. Hepatic levels of reduced glutathione (GSH) and glutathione peroxidase-4 (GPX4) were estimated using ELISA kits from CUSABIO (Houston, United States, cat no. CSB-E12144r, CSB-EL009869RA, respectively) according to the manufacturers’ instructions, while, hepatic levels of nuclear factor erythroid 2-related factor 2 (Nrf2) was estimated using BT LAB ELISA kit (Shanghai, China, Cat no. E1083Ra).

### 2.10 Assay of lipophagy biomarkers

Serum concentrations of total cholesterol, triglycerides (TGs), and Low-density lipoprotein cholesterol (LDL–C) and were determined using kits from Spinreact (Santa Coloma, Spain, cat no. SP41021, MX41031, and 41023, respectively). Serum levels of very low-density lipoprotein cholesterol (VLDL–C) were calculated using a mathematical formula by dividing TGs by 5. Serum levels of Free fatty acids (FFA) was determined using commercial kits from Cell Biolabs, Inc (CA, United States, cat no STA-618).

Hepatic gene expression of fatty acid binding protein-1 (FABP1) was estimated using PCR as mentioned in Section 2.11. Hepatic level of Peroxisome proliferator activated receptor gamma (PPAR-γ) and Platelet glycoprotein 4 (CD36) were estimated using AssayGenie ELISA kit (Dublin, Ireland, cat no. RTFI01079) and Cusabio ELISA kit (Houston, United States, cat no CSB-EL 004927RA), respectively.

### 2.11 Assay of nuclear receptor coactivator 4 (NCOA-4) as a ferritinophagy biomarker

The hepatic levels of NCOA-4 were determined using Mybiosource ELISA kit (California, United States, MBS9905618). In addition, hepatic gene expression levels of NCOA-4 were estimated by using PCR method as discussed in detail in Section 2.15.

### 2.12 Assay of autophagy biomarkers

Serum calcium was measured using the Spinreact kit (Santa Coloma, Spain, cat no. MD1001065). Beclin-1 hepatic levels were determined using the Cusabio ELISA kit (Houston, United States, cat no. CSB-EL002658RA). Hepatic levels of calcineurin-A were assessed using the AssayGenie ELISA kit (Dublin, Ireland, cat no. RTDL00171). In addition, the hepatic gene expression of calcineurin-A was measured by PCR as mentioned in Section 2.15. Hepatic expression of autophagy-related protein light chain 3 (LC3)-II and sequestosome 1 (SQSTM1/p62) were evaluated using immunohistochemical evaluation as described in details in Section 2.14.

### 2.13 Assay of inflammation biomarkers

Hepatic levels of transforming growth factor-B1 (TGF-B1), NF-κB p65 (Phospho-Ser536) and Interleukin-6 (IL-6) were estimated using ELISA kits obtained from (PicoKine C3RA, United States, cat no. EK0514), (MyBioSource, San Diego, CA, United States Cat. No. MBS9511033), and (R&D systems, MN, United States, cat no. R6000B), respectively.

### 2.14 Assessment of transforming growth factor-B (TGF-B1), and Interleukin-6 (IL-6), autophagy-related protein light chain 3 (LC3)-II and sequestosome 1 (SQSTM1/p62) hepatic levels by immunohistochemical evaluation

Paraffin-embedded sections were deparaffinized with xylene and dehydrated in decreasing concentrations of ethanol. Sections were treated with 3% hydrogen peroxide in methanol for 10 min to block endogenous peroxidase activity. For antigen retrieval, tissue sections were processed in EDTA and incubated for 1 h at 37 °C with antibodies against TGF-β1, IL-60, LC3-II and p62 (H-60, Santa Cruz, CA, United States). The best dilution ratio (1/100) was determined in multiple experiments, and all procedures were performed at room temperature according to the manufacturer’s instructions using the HRP-DAB detection system. With the help of 3,3′-diaminobenzidine, binding was visualized, and after counterstaining with hematoxylin, slides were examined. Sections were examined by light microscope using a high objective for TGF-β1, IL-6, LC3-II and p62 positive cells. The percentage of positive areas was investigated in 3 slices per group via ImageJ analysis software. The average percentage was computed.

### 2.15 Assessment of FTH1, calcineurin-A, NCOA-4 and FABP1 using real time polymerase chain reaction (RT-PCR)

The SV total RNA isolation system (Promega Corporation, WI, United States) was used to extract RNA from liver samples in accordance with the manufacturer’s instructions. SensiFAST™ cDNA Synthesis Kit (Meridian Life Science Inc., United States, cat no. BIO-98002) was utilized for reverse transcription of extracted RNA. SensiFAST SYBR® No-ROX Kit (Meridian Life Science Inc., United States) was utilized for performing PCR. The prepared reaction mix samples were applied in real-time PCR using Step One Real-Time PCR (Applied Bio systems, California, United States). The mRNA levels of FTH1, calcineurin-A, NCOA-4, and FABP1 were normalized relative to glyceraldehyde-3-phosphate dehydrogenase (GAPDH) in the same sample. The used primers are mentioned in Table 1.

**TABLE 1:** Experimental design to investigate the impact of EMPA on DEXA-induced hepatic and vascular alterations.

Groups		Days													
		1	2	3	4	5	6	7	8	9	10	11	12	13	14
1	CONTROL	ϴ	ϴ	ϴ	ϴ	ϴ	ϴ	ϴ	ϴ●	ϴ ●	ϴ ●	ϴ ●	ϴ ●	ϴ ●	End
3	DEXA	ϴ	ϴ	ϴ	ϴ	ϴ	ϴ	ϴ	ϴ 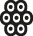	ϴ 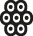	ϴ 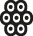	ϴ 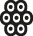	ϴ 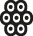	ϴ 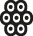	
4	EMPA 10/DEXA	▼	▼	▼	▼	▼	▼	▼	▼ 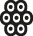	▼ 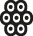	▼ 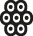	▼ 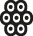	▼ 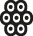	▼ 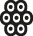	
5	EMPA 30/DEXA	▲	▲	▲	▲	▲	▲	▲	▲ 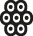	▲ 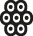	▲ 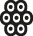	▲ 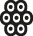	▲ 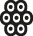	▲ 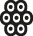	

ϴ: Vehicle: 0.5% CMC orally.

●: 0.9%Normal saline intraperitoneal (i.p.).

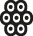
: Dexamethasone (8 mg/kg, i.p.).

▼: Empagliflozin (10 mg/kg, orally).

▲: Empagliflozin (30 mg/kg, orally).

**TABLE 2:** Forward and reverse primers for PCR.

Primer	Forward	Reverse
FTH1	ATC​AAC​CGC​CAG​ATC​AAC​CT	TCT​CCC​AGT​CAT​CAC​GGT​CA
Calcineurin-A	AGA​TGG​ATT​TGA​CGG​AGC​CAC	GCTGCTATTACTGCCGTTGC
NCOA-4	AGTGTCTGGGTCGGTCCAA	GTG​AAT​CTG​AGC​TTT​CAC​CTC​TCG​T
FABP-1	CTG​GGG​AAA​AGG​TCA​AGG​CAG	TTG​TAG​ACG​ATG​TCA​CCC​AGT​G
GAPDH	TCTTCTTGTGCAGTGCCAGC	TGC​CGT​TGA​ACT​TGC​CGT​GG

### 2.16 Statistical analysis

Quantitative data was analyzed and presented by use of mean ± standard error of mean (SEM). Statistical analysis was performed using the GraphPad Prism 9.0 (GraphPad Software Inc. V 9.0.2, San Diego, CA, United States). To compare the scores of the study groups parametrically, one-way analysis of variance (ANOVA) test followed by Tukey’s *post hoc* test was used. While non-parametric results were analyzed using Kruskal-wallis test followed by Dunn’s multiple comparison. Statistical significance was defined as a *p*-value of *<0.05*.

## 3 Results

### 3.1 Effect of EMPA administration on DEXA-induced alteration in metabolic parameters

**FIGURE 1:**
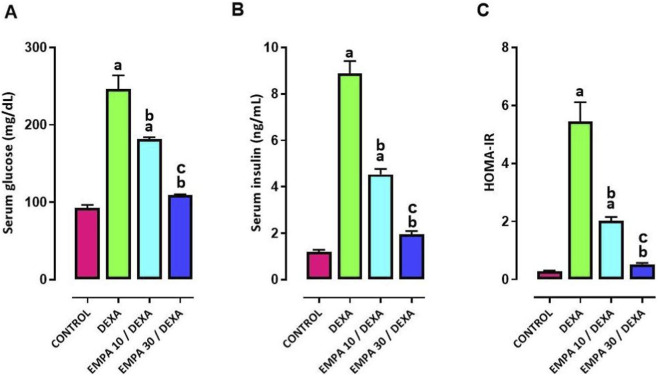
Impact of EMPA on DEXA induced changes in metabolic biomarkers. **(A)** Serum Fasting Glucose, **(B)** Serum Fasting Insulin, **(C)** HOMA-IR. Data were expressed as means ± SEM (n = 4). Mean values were compared using one-way ANOVA followed by *post hoc* Tukey’s multiple comparison test. ^a^significance vs. CONTROL group, ^b^significance vs. DEXA group, ^c^vs. EMPA10/DEXA group. DEXA: Dexamethasone, EMPA: Empagliflozin, HOMAIR: Homeostatic Model Assessment of Insulin Resistance.

Serum levels of fasting blood glucose and fasting insulin were markedly elevated, accompanied by an increased HOMA-IR index, following DEXA administration, indicating metabolic dysfunction. Treatment with both doses of EMPA significantly decreased fasting glucose and insulin levels and improved HOMA-IR (Figures 1A–C).

### 3.2 Effect of EMPA administration on DEXA-induced alteration in liver function

**FIGURE 2:**
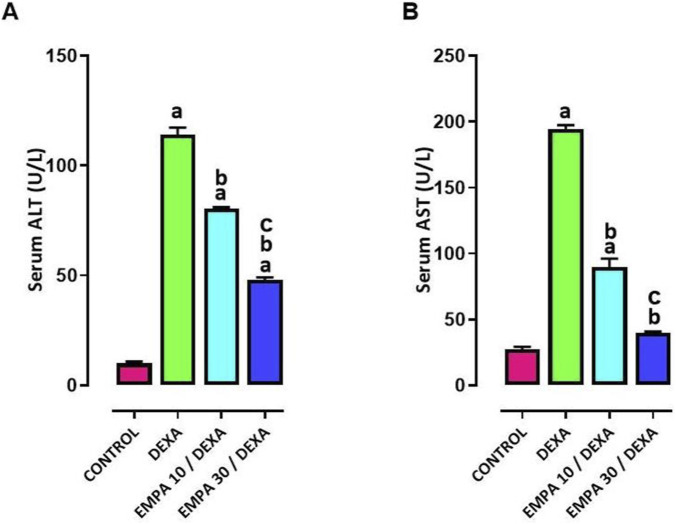
Impact of EMPA on DEXA induced changes in liver function biomarkers. **(A)** Serum ALT, **(B)** Serum AST. Data were expressed as means ± SEM (n = 4). Mean values were compared using one-way ANOVA followed by *post hoc* Tukey’s multiple comparison test. ^a^significance vs. CONTROL group, ^b^significance vs. DEXA group, ^c^vs. EMPA10/DEXA group. DEXA: Dexamethasone, EMPA: Empagliflozin, ALT: alanine aminotransferase, AST: aspartate aminotransferase.

Serum levels of ALT and AST were significantly increased in the DEXA group compared to control group. For the 30 mg/kg dose, EMPA effectively reduced liver enzyme levels that were nearly touch control values. The smaller EMPA dose (10 mg/kg) meaningly lessened serum ALT and AST levels, but to a lesser extent, (Figures 2A,B).

### 3.3 Effect of EMPA administration on DEXA-induced histopathological alterations

**FIGURE 3:**
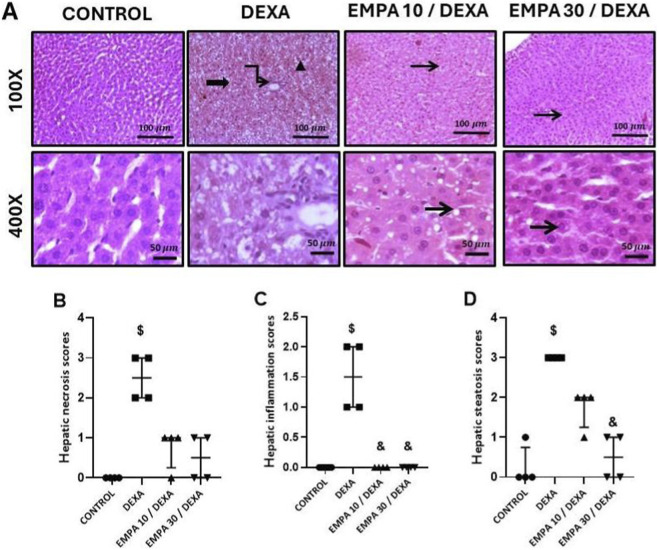
Impact of EMPA on DEXA-induced histopathological changes. **(A)** Representative photomicrograph of liver sections stained with H&E stain from different treatment groups, **(B)** Necrosis score, **(C)** Inflammation score, **(D)** Steatosis score. Thin arrow = steatosis, thick arrow = necrosis, arrowhead = hydropic degeneration, twisted arrow = fibrosis with inflammation. Image magnification = ×100, ×400. Data were expressed as medians (n = 4). ^$^significance vs. CONTROL group, ^&^significance vs. DEXA group Data was expressed as median ± interquartile range, median values were compared using Kruskal–Wallis followed by Dunn’s Multiple comparison test.

As shown in Figure 3A, hepatic sections stained with H&E stain revealed regular histological appearance of hepatic parenchyma in control group. Alternatively, the DEXA- injected group displayed severe necro steatohepatitis characterized by diffuse intracytoplasmic micro vesiculation, hydropic degeneration admixed with scattered hepatocellular necrosis and mild to moderate inflammation admixed with few fibrosis.

Sections from the group received EMPA pretreatment (10 mg/kg) exhibited moderate steatosis with scattered micro and macro vacuoles inside the cytoplasm of hepatocytes in addition to the presence of scattered necrotic hepatocytes. Worth mentioning that the EMPA (30 mg/kg) group exhibited the typical architecture of hepatic parenchyma with occasional single cell death and few intracytoplasmic tiny vacuoles.

Figures 3B-D displayed substantially superior records of liver necrosis, inflammation and steatosis in the DEX- treated rats matched to the control, but obviously lesser in the treatment groups, with minute differences amongst them.

### 3.4 Effect of EMPA management on DEXA-provoked hepatic ultrastructure variations using transmission electron microscopy

**FIGURE 4:**
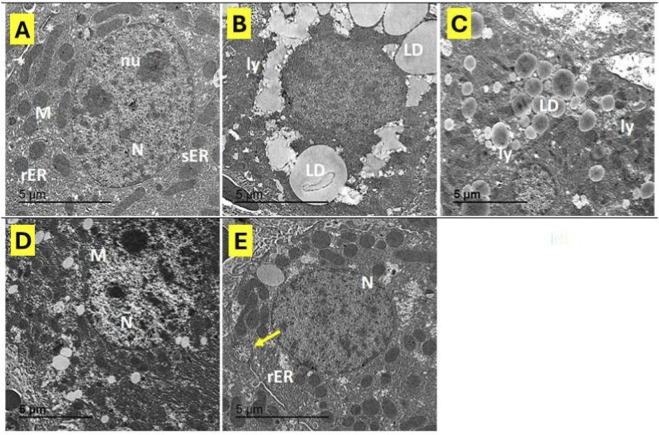
Impact of EMPA on DEXA-induced hepatic ultrastructural changes. Transmission electron micrographs of hepatocytes. **(A)** label refers to Control, **(B,C)** labels refer to DEXA group, **(D)** refers to EMPA10/DEXA & **(E)** refers to EMPA30/DEXA. N: nucleus, nu: nucleolus, M: mitochondria, rER: rough endoplasmic reticulum, sER: smooth endoplasmic reticulum, LD: lipid droplets, Ly: lysosomes, *: bile canaliculus with microvilli, yellow arrows: cell junctions. Magnification: ×10,000 and ×20,000.

As revealed in Figure 4, transmission electron micrographs of hepatocytes from the control group showed normal hepatocyte with euchromatic nucleus of regular outline and prominent nucleolus. The cytoplasm contains numerous mitochondria, multiple arrays of rough endoplasmic reticulum, and vesicles of smooth endoplasmic reticulum. Part of two adjacent hepatocytes enclosing a bile canaliculus with microvilli protruding into its lumen and bounded by desmosomes (Figure 4A). In contrast, hepatocytes from the DEXA-treated group exhibited marked ultrastructural alterations, the hepatocytes exhibited both large and small lipid droplets, numerous electron-dense lysosomes, dilated rough endoplasmic reticulum, and mitochondria of varying sizes. Additionally, bile canaliculi showed distorted microvilli and poorly defined intercellular junctions. The hepatocyte nuclei appeared small, containing clusters of heterochromatins (Figures 4B,C). Treatment with EMPA at 10 mg/kg partially ameliorated DEXA-induced changes, further dilation and fragmentation of the rough endoplasmic reticulum were still evident, but with less cytoplasmic vacuolization compared to the diseased group (Figure 4D). Meanwhile, hepatocytes from rats treated with EMPA at 30 mg/kg displayed nearly normal ultrastructure, the hepatocytes displayed a normal morphology and structural organization, with evenly distributed rough endoplasmic reticulum, a typical mitochondrial pattern and cell junction (Figure 4E). These findings indicate a dose-dependent protecting consequence of EMPA versus DEXA-provoked hepatic harm at the subcellular level.

### 3.5 Effect of EMPA administration on DEXA-provoked alterations in iron homeostasis

**FIGURE 5:**
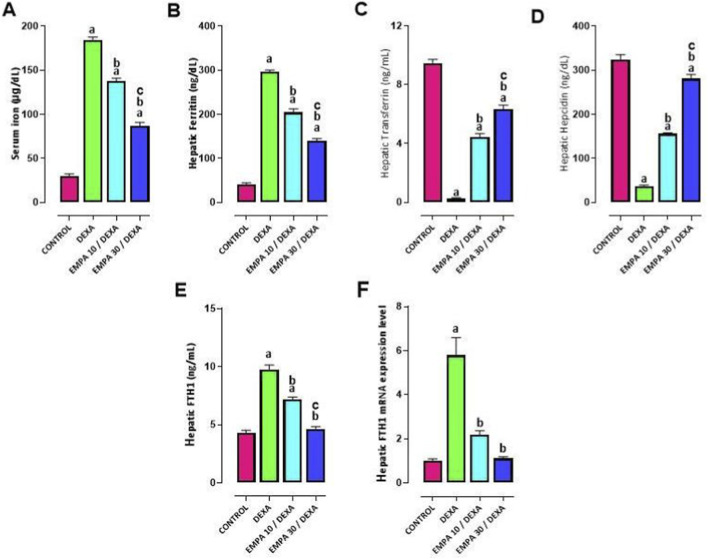
Impact of EMPA on DEXA-induced changes in iron homeostasis biomarkers. **(A)** Serum iron, **(B)** Hepatic Ferritin, **(C)** Hepatic Transferrin **(D)** Hepatic Hepcidin, **(E)** Hepatic FTH1, **(F)** FTH1 mRNA expression. Data were expressed as means ± SEM (n = 4). Mean values were compared using one-way ANOVA followed by *post hoc* Tukey’s multiple comparison test. ^a^significance vs. CONTROL group, ^b^significance vs. DEXA group, ^c^significance vs. EMPA10/DEXA group. DEXA: Dexamethasone, EMPA: Empagliflozin, FTH1: Ferritin heavy chain.

DEXA received rats showed a significant increase in serum iron compared to control group. While rats received both doses of EMPA showed marked decreases in serum iron (Figure 5A). Hepatic biochemical parameters of iron homeostasis were included: ferritin, transferrin, hepcidin, and FTH1. Rats administered DEXA showed higher levels of hepatic ferritin and mRNA expression of FTH1, and lower levels of hepatic transferrin, hepcidin as matched to the control group (Figures 5B–F). EMPA pre-administration (10 and 30 mg/kg) significantly decreased hepatic ferritin, gene and protein expression of FTH, and upregulated hepatic transferrin and hepcidin levels–dose dependently, compared to the DEXA group (Figures 5B–F).

Generally, EMPA effectively reversed DEXA-induced hepatic disrupted iron homeostasis in a dose dependent manner, supporting its protective potential.

### 3.6 Effect of EMPA application on DEXA-provoked alterations in hepatic oxidative stress

**FIGURE 6:**
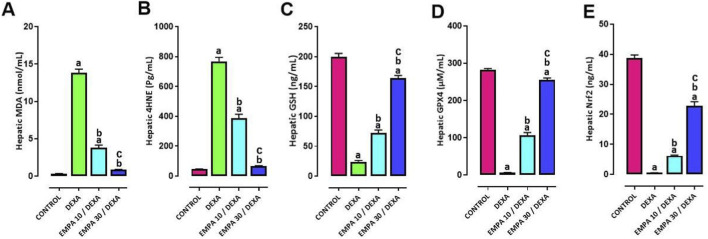
Impact of EMPA on DEXA-induced changes in hepatic oxidant/antioxidant biomarkers. **(A)** MDA, **(B)** 4HNE, **(C)** GSH, **(D)** GPX4, **(E)** Nrf2. Data were expressed as means ± SEM (n = 4). Mean values were compared using one-way ANOVA followed by *post hoc* Tukey’s multiple comparison test. ^a^significance vs. CONTROL group, ^b^significance vs. DEXA group, ^c^significance vs. EMPA10/DEXA group. DEXA: Dexamethasone, EMPA: Empagliflozin, 4HNE: 4-Hydroxy-2-nonenal, MDA: Malondialdehyde, GSH: Glutathione, GPX4: Glutathione Peroxidase-4, Nrf2: Nuclear Factor Erythroid 2–Related Factor 2.

Rats received DEXA, developed substantial oxidative stress in liver tissue, which displayed high lipid peroxidation levels, while their antioxidant resource levels decreased. Rats administered DEXA showed elevated MDA and 4-HNE hepatic levels compared to control rats (Figures 6A,B). The liver redox equilibrium showed significant deterioration by DEXA treatment, evident by a significant reduction in GSH, GPX4 and Nrf2 hepatic levels (Figures 6C–E). The administration of EMPA markedly ameliorated oxidant/antioxidant status, with a more significant effect of EMPA (30 mg/kg) than EMPA (10 mg/kg), matched to the DEXA group (Figures 5E, 6A).

### 3.7 Effect of EMPA administration on DEXA-provoked alterations in the ferritinophagy

**FIGURE 7:**
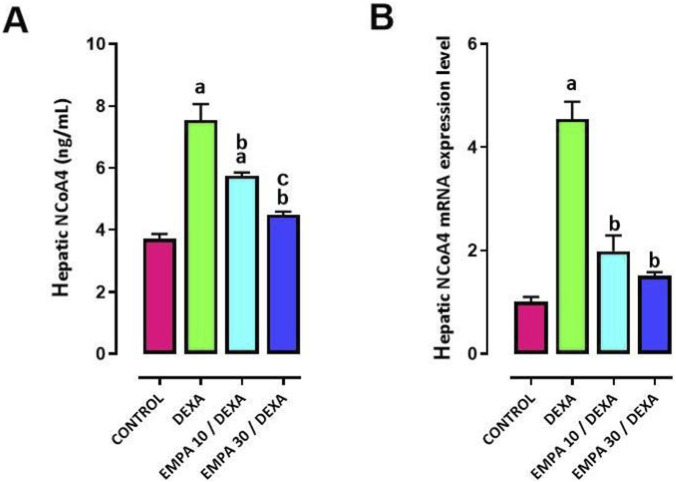
Impact of EMPA on DEXA-induced changes in hepatic protein and gene expression of NCOA4. **(A)** Hepatic NCOA4, **(B)** NCOA4 mRNA expression. Data were expressed as means ± SEM (n = 4). Mean values were compared using one-way ANOVA followed by *post hoc* Tukey’s multiple comparison test. ^a^significance vs. CONTROL group, ^b^significance vs. DEXA group. DEXA: Dexamethasone, EMPA: Empagliflozin, NCOA4: Nuclear Receptor Coactivator-4.

DEXA significantly induced the hepatic gene and protein expression of NCOA4, a ferritinophagy biomarker, compared to control group (Figure 7A,B). The administration of EMPA at both doses profoundly declined the gene and protein expression levels of NCOA4, competed to the DEXA group (Figures 7A,B).

### 3.8 Effect of EMPA application on DEXA-provoked alterations in autophagy

**FIGURE 8:**
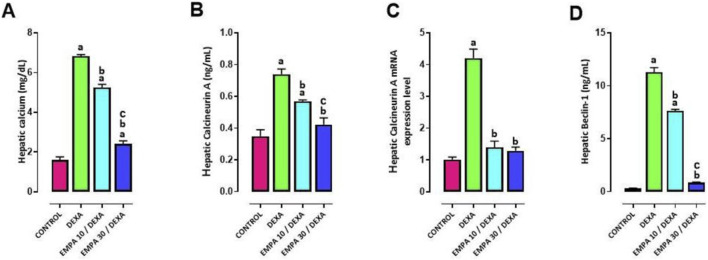
Impact of EMPA on DEXA-induced changes in autophagy biomarkers. **(A)** Serum Calcium **(B)** Hepatic Calcineurin A **(C)** Calcineurin A mRNA expression, **(D)** Hepatic Beclin-1. Data were expressed as means ± SEM (n = 4). Mean values were compared using one-way ANOVA followed by *post hoc* Tukey’s multiple comparison test. ^a^significance vs. CONTROL group, ^b^significance vs. DEXA group, ^c^significance vs. EMPA10/DEXA group. DEXA: Dexamethasone, EMPA: Empagliflozin.

DEXA installation showed a significant increase in each of serum calcium, hepatic gene and protein expression of calcineurin A, and hepatic levels of beclin-1, when compared to the control group (Figures 8A–D). The administration of EMPA significantly modulated these autophagic biomarkers in a dose dependent manner, evident by the significant decrease of serum calcium, hepatic gene and protein expression of calcineurin A, and hepatic levels of beclin-1 in comparison to the DEXA group (Figures 8A–D).

**FIGURE 9:**
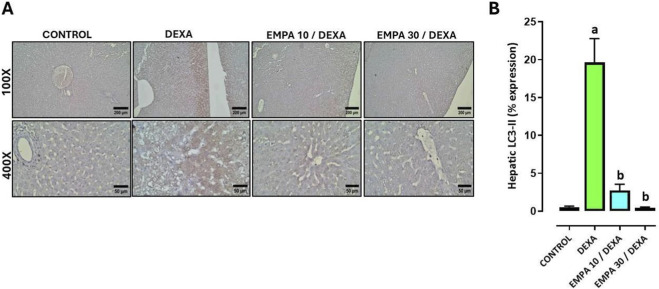
Impact of EMPA on DEXA-induced changes in hepatic LC3-II expression. **(A)** Photomicrograph of immunostained liver sections against LC3-II, **(B)** LC3 II expression. Data were expressed as means ± SE (n = 4). Mean values were compared via one-way ANOVA followed by *post hoc* Tukey’s multiple comparison test. ^a^significance vs. CONTROL group, ^b^significance vs. DEXA group. DEXA: Dexamethasone, EMPA: Empagliflozin, LC3-II: autophagy-related protein light chain 3.

**FIGURE 10:**
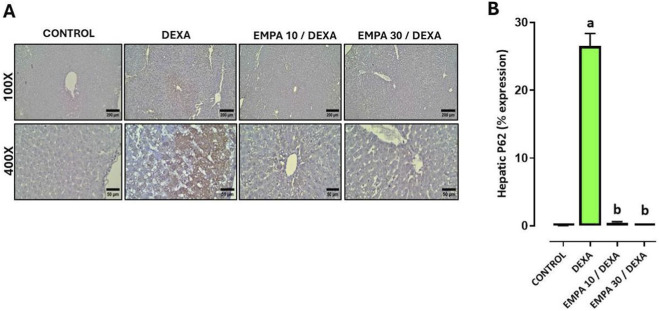
Impact of EMPA on DEXA-induced changes in hepatic p62 expression. **(A)** Photomicrograph of immunostained liver sections against p62, **(B)** p62 expression. Data were expressed as means ± SE (n = 4). Mean values were compared via one-way ANOVA followed by *post hoc* Tukey’s multiple comparison test. ^a^significance vs. CONTROL group, ^b^significance vs. DEXA group. DEXA: Dexamethasone, EMPA: Empagliflozin, p62: sequestosome 1.

Additionally, the expression of LC3-II and p62 in hepatic sections of different treatment groups revealed that the control group show a negative cytoplasmic expression. Conversely, DEXA-administrated group exhibits diffuse strong brownish cytoplasmic expression. In contrast, EMPA-treated groups show a dose-dependent reduction in p62 immunoreactivity, as the low dose-treated group shows marked reduction in cytoplasmic staining reaction, the high dose-treated group shows remarkably restored the expression to near-normal levels (Figures 9A, 10A).

Figures 9B, 10B demonstrated that DEXA caused a significant increase in LC3-II and p62 hepatic expressions. However, EMPA administration caused a dose-dependent decrease in both LC3-II and p62 expressions comparing to the DEXA group.

### 3.9 Effect of EMPA application on DEXA-generated deviations in lipophagy

**FIGURE 11:**
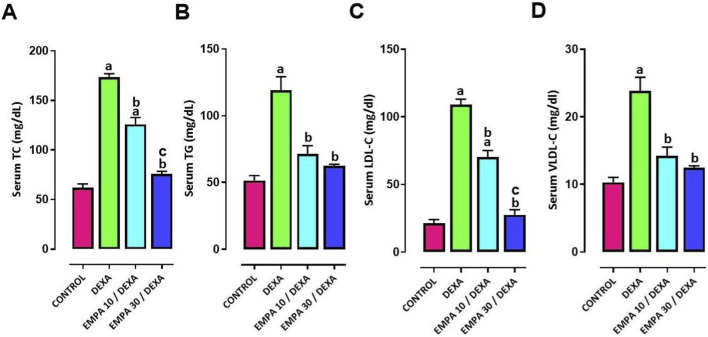
Impact of EMPA on DEXA induced changes in serum lipid profile of DEXA-injected rats. **(A)** Serum TC, **(B)** Serum TG, **(C)** serum LDL-C, **(D)** serum VLDL-C, **(E)** serum HDL-C. Data were expressed as means ± SEM (n = 4). Mean values were compared using one-way ANOVA followed by *post hoc* Tukey’s multiple comparison test. ^a^significance vs. CONTROL group, ^b^significance vs. DEXA group, ^c^significance vs. EMPA10/DEXA group. DEXA: Dexamethasone, EMPA: Empagliflozin, TC: total cholesterol, TG: triglycerides, LDL-C: low density lipoprotein cholesterol, VLDL-C: very low-density lipoprotein cholesterol, HDL-C: high-density lipoprotein cholesterol.

The effect of DEXA administration on lipid profile was found to be significant since serum TG, TC, LDL-C, and VLDL-C serum levels showed marked increases, in comparison to control group. Attempts to mitigate these changes were made via EMPA. Both doses of EMPA resulted in a noteworthy lessening of TG, TC, LDL-C, and VLDL-C serum levels in comparison to DEXA group. The effect of the higher dose was more profound on reducing serum levels of TC and LDL-C (Figures 11A–D).

**FIGURE 12:**
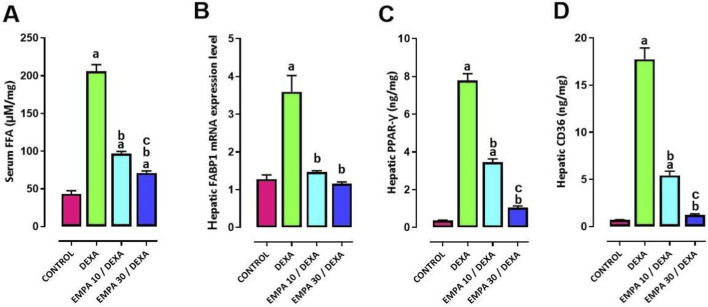
Impact of EMPA on DEXA induced changes in lipophagy biomarkers. **(A)** serum FFA, **(B)** hepatic FABP1, **(C)** hepatic PPAR-γ, **(D)** hepatic CD36. Data were expressed as means ± SEM (n = 4). Mean values were compared using one-way ANOVA followed by *post hoc* Tukey’s multiple comparison test. ^a^significance vs. CONTROL group, ^b^significance vs. DEXA group, ^c^significance vs. EMPA10/DEXA group. DEXA: Dexamethasone, EMPA: Empagliflozin, FFA: free fatty acids, FABP1: Fatty Acid-Binding Protein-1, PPAR-γ: Peroxisome Proliferator-Activated Receptor Gamma, CD36: Cluster of Differentiation 36.

DEXA-induced lipid dysregulation was further confirmed by the significant increase in serum FFA, hepatic mRNA expression level of FABP1 and hepatic levels of both PPAR-γ and CD36, competed to control group. On the contrary, EMPA (10 and 30 mg/kg) application meaningfully ameliorated lipid dysregulation as displayed by the diminution in serum FFA, hepatic gene expression level of FABP1 and hepatic levels of both PPAR-γ and CD36 with a more significant effect of EMPA (30 mg/kg) on reducing PPAR-γ and CD36 hepatic levels (Figures 12A–D).

### 3.10 Effect of EMPA administration on DEXA-provoked alterations in inflammation

**FIGURE 13:**
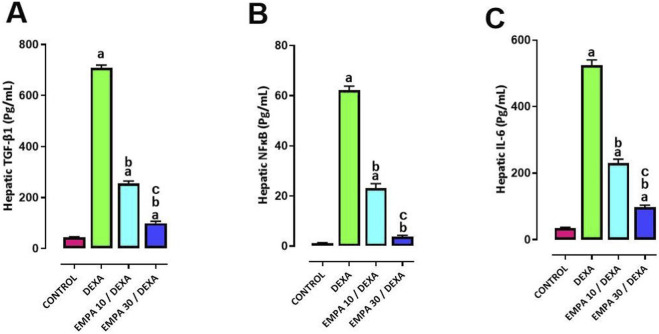
Impact of EMPA on DEXA-induced changes in inflammation biomarkers. **(A)** TGF-β1, **(B)** NF-κB, **(C)** IL-6. Data were expressed as means ± SEM (n = 4). Mean values were compared using one way ANOVA followed by *post hoc* Tukey’s multiple comparison test. ^a^significance vs. CONTROL group, ^b^significance vs. DEXA group, ^c^significance vs. EMPA10/DEXA group. DEXA: Dexamethasone, EMPA: Empagliflozin, TGF-β1: Transforming Growth Factor Beta 1, NF-κB: Nuclear Factor Kappa-light-chain-enhancer of Activated B Cells, IL-6: Interleukin 6.

Rats administered DEXA showed considerable rise in the hepatic level of TGF-β1, NF-κB and IL-6 (Figures 13A–C) compared to the control group. In a dose-related manner, the administration of EMPA significantly decreased the protein levels of TGF-β1, NF-κB, and IL-6, compared to the DEXA group.

**FIGURE 14:**
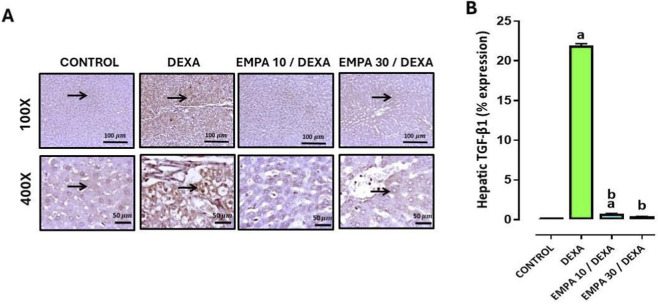
Impact of EMPA on DEXA-induced changes in hepatic TGF-β1 expression. **(A)** Photomicrograph of immunostained liver sections against TGF-β1, **(B)** TGF-β1 expression. Thin arrow indicates positively immunostained hepatocytes. Data were expressed as means ± SE (n = 4). Mean values were compared via one-way ANOVA followed by *post hoc* Tukey’s multiple comparison test. ^a^significance vs. CONTROL group, ^b^significance vs. DEXA group DEXA: Dexamethasone, EMPA: Empagliflozin, TGF-B: Transforming growth factor beta.

Furthermore, the expression of TGF-β1 in hepatic sections of different treatment groups confirmed that the control group showed negative to occasional weakly stained hepatocytes, the DEXA group showed strong immunopositivity, EMPA 10 group showed no staining, and EMPA 30 showed occasional immunostaining against TGF-β1 (Figure 14A).

**FIGURE 15:**
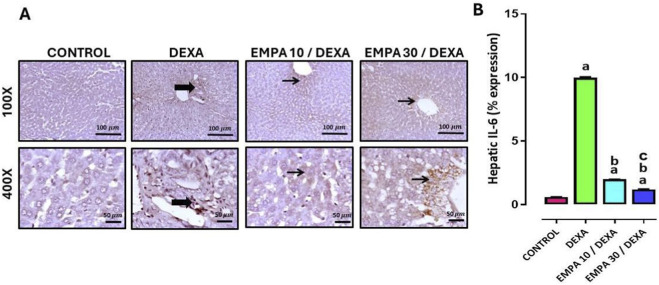
Impact of EMPA on DEXA-induced changes in hepatic IL-6 expression. **(A)** Photomicrograph of immunostained liver sections against IL-6, **(B)** IL-6 expression. Thin arrow indicates positive stained hepatocytes, thick arrow indicates positive inflammatory cells. Data were expressed as means ± SE (n = 4). Mean values were compared via one-way ANOVA followed by *post hoc* Tukey’s multiple comparison test. ^a^significance vs. CONTROL group, ^b^significance vs. DEXA group, ^c^significance vs. EMPA10/DEXA group. DEXA: Dexamethasone, EMPA: Empagliflozin, IL-6: Interleukin-6.

Figure 14B demonstrated that DEXA caused a significant increase in TGF-β1 expression. However, EMPA administration caused a substantial lessening in TGF-β1 expression comparing to the DEXA group. The expression of IL-6 in hepatic tissue showed that the control group exhibited no staining positivity. The DEXA group showed mild to moderate immunopositivity and positive staining for inflammatory cells, the EMPA 10 group showed negative to a few weak immunopositivity, and EMPA 30 group showed mild immunopositivity (Figure 15A).

Figure 15B showed that the hepatic expression of IL-6 was markedly increased in the disease group relative to the control group (P < 0.05). While EMPA (10 or 30 mg/kg) administration significantly provoked IL-6 hepatic expression, compared to the diseased group.

The expression of IL-6 in hepatic tissue showed that the control group exhibited no staining positivity (Figures 15A,B). The DEXA group showed mild to moderate immunopositivity and positive staining for inflammatory cells, the EMPA 10 group showed negative to a few weak immunopositivity, and EMPA 30 group showed mild immunopositivity. The hepatic expression of IL-6 was markedly increased in the disease group relative to the control group (P < 0.05). While EMPA (10 or 30 mg/kg) administration significantly provoked IL-6 hepatic expression, compared to the diseased group.

## 4 Discussion

Non-alcoholic fatty liver disease (NAFLD) is a prevalent hepatic disorder worldwide, encompassing conditions from simple steatosis (NAFL) to non-alcoholic steatohepatitis (NASH) which involve inflammation and fibrosis can advance to severe liver injury and multiple complications (Friedman et al., 2018). Currently, no approved pharmacological treatment exists for NAFLD, particularly in patients with coexisting T2DM. SGLT-2 inhibitors, a new class of antidiabetic agents, have shown potential in reducing hepatic lipid accumulation and inflammation (Garvey et al., 2018). Nonetheless, the precise mechanisms underlying these beneficial effects remain unclear.

Insulin resistance (IR) plays a key role in both the onset and advancement of NAFLD. Treatment with DEXA has been reported to promote the induction of IR (Patel et al., 2011; Su et al., 2014; Williams et al., 2012) and NAFLD in numerous reports (El-Sonbaty et al., 2019; Vinodraj et al., 2015). DEXA induced adverse metabolic consequences like change in insulin sensitivity, presence of dyslipidemia, and hepatic steatosis, which resemble the development of NASH, have been depicted in both human and various experimental models (Peng et al., 2020). In our study glucose hemostasis and IR were assessed and significant elevation of glucose, insulin and HOMA-IR in DEXA group that were markedly lessened upon EMPA application.

The capacity of DEXA to provoke these pathological alterations within a relatively short duration represents an additional reason for its selection (Korach-André et al., 2005; Kumar et al., 2015; Okumura et al., 1998). In the present study, DEXA administration led to the manifestation of NASH-like pathological features, accompanied by disturbances in hepatic functional biomarkers and multiple metabolic disorders, such as hyperglycemia, hypercholesterolemia, and hypertriglyceridemia. Our study confirms these findings by significant increase in serum ALT and AST levels indicating hepatic injury that was further confirmed by histopathological evaluation and electron microscopy upon DEXA. Previous effects were dose dependently reversed upon EMPA application. EMPA’s ability to protect against liver damage was previously confirmed in rat and mouse model of liver injury of methotrexate-induced hepatotoxicity (Kalantari et al., 2024; El-Kashef and Sewilam, 2023), and in a mouse model of ethanol-induced liver injury (Abdelhamid et al., 2020).

Iron overload enhances the generation of reactive oxygen species (ROS), leading to cellular dysfunction or death, tissue injury, and disease development (Dixon et al., 2012; Tian et al., 2020). Moreover, excessive iron triggers ferroptosis—an iron-dependent, recently identified form of regulated cell death, which has been implicated in the progression of NASH pathology (Dixon et al., 2012; Tsurusaki et al., 2019). We noticed marked alteration in iron homeostasis in the DEXA group evident by elevated serum iron hepatic ferritin levels, and hepatic FTH1 level and expression with diminution of hepatic transferrin and hepcidin levels. This observed imbalance aggravated ROS production via the Fenton reaction, aggravated ferroptosis and oxidative stress (Anandhan et al., 2023; Dixon et al., 2012; Liang et al., 2019; Lu et al., 2021; Tian et al., 2020; Xie et al., 2016), as reflected by the elevation of hepatic MDA and 4-HNE with diminished hepatic GSH, GPX4, and NRF2 levels. A better effect seen with the larger EMPA dose, indicating shifting the cells back to an iron homeostasis state and relieving iron build-up, an effect that may explain the subsequent mitigation of oxidative stress, ferroptosis, autophagy, and inflammation.

A former study demonstrated a close relation between ferroptosis and autophagy, especially ferritinophagy and lipophagy (Li N. et al., 2020). Different studies demonstrated that ferritin degradation by ferritinophagy can enhance ferroptosis in various tumor cell lines (Hou et al., 2016; Gao et al., 2016). Ferritinophagy, a recently identified ferritin autophagic pathway, mediates the regulation of iron homeostasis in cooperation with NCOA4 (Mancias et al., 2014). The interaction between FTH1 and NCOA4 has been proposed as a potential mechanism contributing to the incidence of ferroptosis (Fang et al., 2021). NCOA4 selectively binds FTH1 in the autophagosome and delivers it into the lysosome, resulting in iron release (Del Rey and Mancias, 2019; Santana-Codina and Mancias, 2018). Our results came to confirm these previous studies. DEXA led to a significant increase in hepatic levels and expression of NCOA4 and FTH1 indicating the activation of ferritinophagy and subsequent ferroptosis activation. Novelly, in our study, EMPA administration at low and high doses markedly decreased NCOA4 and FTH1 mRNA expression and protein levels, indicating suppression of ferritinophagy and ferroptosis. The elevation in Ca^+2^ linked with iron overload and previous studies further confirm activation of autophagy via TFEB nuclear translocation (an autophagy-promoting factor), as supported by raised Beclin-1 levels (Nakamura et al., 2009) and calcineurin activity (Klee et al., 1979; Rusnak and Mertz, 2000). Our DEXA-treated rats exhibited elevated Ca^+2^ and calcineurin activity, which is consistent with the results of previous studies obtained by de Salvi Guimarães et al. (de Salvi Guimarães et al., 2017). Previous studies have demonstrated that the activation capability of DEXA on the AMP-activated protein kinase (AMPK) signaling pathway is contingent on Glucocorticoid receptor α (Zhan et al., 2021). The AMPK signalling pathway is intricately linked to autophagy-dependent ferroptosis. AMPKα-mediated Beclin-1 phosphorylation triggers LC3-II puncta accumulation and autophagy, potentially promoting ferroptosis (Song et al., 2018). This activation of autophagy, in turn, led to lipid peroxidation and iron accumulation as part of the response to the induction of ferroptosis (Lu et al., 2025). Our data strongly supports these findings, as the autophagy protein levels were significantly upregulated in the DEXA group, highlighted by the elevated Beclin-1, LC3 II, and p62 hepatic levels; such increase in the autophagic biomarkers was significantly mitigated by treatment with EMPA, and this effect was previously confirmed by several studies (Wang C.-C. et al., 2022; Qusty et al., 2025; Madonna et al., 2023).

Lipophagy is a form of selective autophagy that specifically targets and degrades lipid droplets (LDs), playing a crucial role in maintaining their homeostasis. Elevated plasma free fatty acid (FFA) levels result from adipose tissue lipolysis, which serves as the primary contributor to triglyceride (TG) accumulation in the liver (Utzschneider and Kahn, 2006). This upsurge consequently causes lipotoxicity, insulin resistance, oxidative stress and inflammation, which play key roles in NASH. Administration of DEXA led to the lipid metabolism disruption (Nguelefack-Mbuyo et al., 2022; Alona et al., 2021) with increased levels of serum FFAs, hepatic FABP-1, PPAR-γ and CD36 expression reflecting acute dyslipidemia, metabolic disturbance and inflammation. CD36 is a multifunctional protein that plays a crucial role in facilitating the transmembrane transport of long-chain fatty acids (Hao et al., 2020). FATPs are primarily responsible for mediating the uptake of long-chain fatty acids across the cell membrane, whereas FABPs are soluble proteins that participate in the intracellular trafficking of fatty acids. However, it remains uncertain whether glucocorticoid-induced hepatic lipid accumulation is linked to the upregulation of FATPs. Given that DEXA is among the most prescribed synthetic glucocorticoids. During lipophagy, LDs are degraded to yield FFAs (Singh and Cuervo, 2012). Lipid accumulation–induced lipotoxicity leads to mitochondrial dysfunction and disruption of insulin signaling (Marchesini et al., 2001). Additionally, elevated hepatic uptake of FFAs, together with insulin resistance and hyperinsulinemia, enhances lipogenesis in individuals with NASH by activating lipogenic enzymes. Within hepatocytes, FFAs are taken up via fatty acid transport protein (FATP) and fatty acid translocase (FAT/CD36), which mediate the transport of long-chain fatty acids (LCFAs) (Wang F. et al., 2022; Lu et al., 2023; Wilson et al., 2016). Once inside the hepatocytes, FFAs bind to FABP-1 and are directed to their destiny dependent on nutritional situations (Wang et al., 2015). Modern findings indicate that selective destruction of hepatic PPAR-γ, a crucial controller of NAFLD (Singh et al., 2025), can influence downstream genes involved in lipid metabolism, thereby decreasing lipid buildup (Xu et al., 2024). Research indicates that inhibition of PPAR-γ leads to reduced CD36 expression (Zhang et al., 2019). Conversely, EMPA treatment, particularly at higher doses, significantly reversed these changes in a dose-dependent manner. This lipid-lowering effect may result from enhanced peripheral utilization of glucose and lipids and improved insulin sensitivity. Supporting this, ElBaset et al. demonstrated that EMPA reduced TG and TC levels in rats with thioacetamide-induced hepatic injury (ElBaset et al., 2022). Also, a former study proved that EMPA ameliorated FFA-induced lipotoxicity in renal proximal tubular cells via the PPAR-γ/CD36 pathway in obese mice (Huang et al., 2021). Collectively, EMPA, by promoting glycosuria and reducing systemic glucose levels, indirectly improves hepatic glucose homeostasis, thereby alleviating insulin resistance in the liver. This metabolic shift decreases *de novo* lipogenesis and alters lipid flux, resulting in reduced hepatic TG accumulation (Sattar et al., 2018). These findings suggest that EMPA may directly target the initial “first hit” of NASH hepatic fat accumulation, thereby mitigating lipotoxic injury and preventing progression to inflammation and fibrosis.

Molecular patterns (DAMPs), activate macrophages through TLR4/NF-κB signaling, inducing pro-inflammatory cytokines such as IL-6 (Bruckbauer et al., 2016). Our data showed increased hepatic NF-κB and IL-6 after DEXA administration, which were significantly suppressed by EMPA. Furthermore, EMPA decreased TGF-β1 expression, reducing hepatic stellate cell activation and fibrosis. These anti-inflammatory and anti-fibrotic effects align with previous studies (Abouzed et al., 2024; Alzokaky et al., 2025; Elsayed et al., 2024; Shentu et al., 2021) supporting EMPA’s role in disrupting the inflammation-fibrosis axis in NASH.

Focusing on hepatic macrophages, it is uncertain whether extra iron directly activates macrophages. The documented characteristics of NASH are hepatic steatosis and inflammation, with or without hepatic fibrosis (Farrell et al., 2018). Severe steatosis increases the accumulation of lipid molecules that are harmful and can cause damage to the liver, such as cholesterol and saturated FFAs (Meng et al., 2022). As a result, hepatocytes release various intracellular components, including damage-associated molecular patterns (DAMPs), which readily interact with TLR4, leading to activation of the downstream IκB/NF-κB signaling pathway, and release of a series of pro-inflammatory cytokines such as IL-6 that ultimately trigger an inflammatory response (Lin et al., 2024; Zhang et al., 2022; Bruckbauer et al., 2016), promoting the progression to NASH. Our study results showed a significant increase in pro-inflammatory markers such as NF-κB and IL-6 after DEXA administration. A direct anti-inflammatory effect of EMPA is strongly supported by the significant inhibition of NF-κB and IL- 6 hepatic protein expression levels. Hepatic stellate cells (HSCs), the primary cells responsible for producing collagen during the process of fibrosis, are activated by TGF-β1, a vital pro-fibrotic cytokine that mediates collagen synthesis (Balta et al., 2015). Moreover, TGF-β1 was stated to provoke the destruction of IκBα, developing in the augmentation of NF-κB (Freudlsperger et al., 2013). The outcomes of the existing investigation revealed that EMPA administration meaningfully decreased the expression level of TGF-β1 in liver tissue comparing to the DEXA group, indicating potent inhibition of HSC motivation and decline the buildup of collagen and alleviation of liver fibrosis. Inhibition of NF-κB inflammation pathway by EMPA was previously confirmed by multiple studies (Abouzed et al., 2024; Alzokaky et al., 2025), while EMPA’a attenuation of fibrosis by downregulation of TGF- β 1 was also multiply mentioned by different studies (Shentu et al., 2021; Elsayed et al., 2024; Huang et al., 2024). These molecular and cellular changes collectively demonstrate EMPA’s ability to disrupt the inflammation-fibrosis axis, a major pathological driver of liver injury and fibrotic progression in NASH. To better contextualize the hepatic findings within the systemic metabolic improvements observed with EMPA treatment, it is important to recognize that EMPA exerts multiple metabolic actions beyond glycemic lowering that are relevant to liver biology. Clinical and translational studies have repeatedly shown that EMPA reduces hepatic steatosis, liver enzymes, and fibrosis indices in patients with type 2 diabetes and metabolic dysfunction–associated steatotic liver disease (MASLD/NAFLD) in conjunction with improvements in insulin sensitivity and reductions in body weight and adiposity. For example, a recent randomized clinical trial demonstrated significant improvements in liver fat content and liver enzyme profiles in empagliflozin-treated patients compared with control, changes that align with reductions in systemic metabolic risk factors such as glucose and insulin levels (Erfanifar et al., 2025). Moreover, mechanistic evidence from animal models indicates that SGLT2 inhibitors, including EMPA, can modulate hepatic lipid metabolism and inflammatory signaling pathways through AMPK activation and reduced lipogenesis, processes that are closely linked to improved systemic insulin sensitivity (Ala, 2021). While some preclinical studies also suggest that SGLT2 inhibitors may enhance markers of autophagy and related signaling in the liver, these effects are context-dependent and often involve upstream metabolic regulators such as AMPK, whose activity is modulated by systemic energy balance and insulin action (Li T. et al., 2020). Taken together, these data support the concept that the hepatic changes observed with empagliflozin are likely a result of an integrated response to global metabolic improvement, including enhanced insulin sensitivity, reduced lipotoxicity, and altered substrate utilization, rather than being solely mediated by a direct hepatocellular action of the drug. While the current findings highlight both systemic and hepatic benefits, the precise contribution of direct hepatic versus secondary systemic effects remains unclear. To clarify these relationships, future studies should incorporate mechanistic approaches, such as experiments in isolated hepatocytes, tissue-specific knockout models, or interventions that separate systemic metabolic improvements from direct hepatic effects. Such studies will help define the extent to which empagliflozin exerts direct hepatoprotective actions versus effects that are downstream of improved overall metabolism.

Limitations of the study: A limitation of this study is the small sample size (n = 4 per group), which was justified using the resource equation approach appropriate for exploratory, hypothesis-generating animal research. As a result, the findings should be interpreted as indicative of trends rather than definitive mechanistic conclusions, and larger confirmatory studies are warranted. In addition, ferroptosis-specific inhibitors were not used, so the involvement of ferroptosis-related pathways is based on associative evidence rather than definitive causal proof. The use of ferroptosis-specific inhibitors will be considered in future studies to confirm pathway specificity. The outcome of EMPA was assessed only in a DEXA-induced NASH model. Though this model mimics the main metabolic and inflammatory characteristics of NASH, it may not entirely characterize the complexity and chronic development of human NASH. Consequently, additional studies utilizing additional diet- or toxin-induced NASH models are necessary to approve the wider pertinency of our findings.

In conclusion, as one of the SGLT-2 inhibitors, EMPA may exert hepatoprotective effects against DEXA-induced NASH not only through metabolic regulation but also by modulating oxidative stress pathways. Emerging evidence indicates that these agents inhibit oxidative ferroptosis, a regulated form of cell death strongly associated with autophagy. In particular, the suppression of ferritinophagy and lipophagy, two selective autophagy processes linked to iron and lipid turnover, appears to mitigate inflammatory signaling and cellular injury. Thus, targeting the interplay between ferroptosis and selective autophagy provides a novel mechanistic insight into how SGLT-2 inhibitors may reduce hepatic inflammation and contribute to the therapeutic management of NASH and related metabolic liver diseases.

It is essential to note that the conclusions of this work validated the marked relation among iron overload, ferroptosis stimulation, autophagy disturbances, lipid metabolism dysregulation, inflammation, and liver injury in the DEXA-provoked NASH model. Nevertheless, these relations do not verify direct causation. Whereas the results advocated potential mechanistic relations, more targeted analyses are required to establish conclusive pivotal relations. Also, SGLT-2 inhibitors need further preclinical and clinical studies.

## Data Availability

The datasets generated during and/or analyzed during the current study are available from the corresponding author on reasonable request.
