# Correction: Empagliflozin attenuates dexamethasone-induced non-alcoholic steatohepatitis by regulation of ferroptosis, inflammation and autophagy

**DOI:** 10.3389/fphar.2026.1835450

**Published:** 2026-04-10

**Authors:** Naif S. Alharbi, Marwa E. Abdelmageed, Manar A. Nader, Marwa S. Serrya

**Affiliations:** 1 Department of Pharmacology and Toxicology, Faculty of Pharmacy, Mansoura University, Mansoura, Egypt; 2 Department of Pharmacology and Toxicology, Faculty of Pharmacy, Mansoura National University, Gamasa, Egypt

**Keywords:** autophagy, dexamethasone, empagliflozin, ferroptosis, inflammation, non-alcoholic steatohepatitis

Author **Naif S. Alharbi** was erroneously assigned to affiliation ‘Department of Pharmacology and Toxicology, Faculty of Pharmacy, Mansoura National University, Gamasa, Egypt’. This affiliation has now been removed for author Naif S. Alharbi.

The original article has been updated.

