# Correction: Empagliflozin attenuates dexamethasone-induced non-alcoholic steatohepatitis by regulation of ferroptosis, inflammation and autophagy

**DOI:** 10.3389/fphar.2026.1874997

**Published:** 2026-05-25

**Authors:** Naif S. Alharbi, Marwa E. Abdelmageed, Manar A. Nader, Marwa S. Serrya

**Affiliations:** 1 Department of Pharmacology and Toxicology, Faculty of Pharmacy, Mansoura University, Mansoura, Egypt; 2 Department of Pharmacology and Toxicology, Faculty of Pharmacy, Mansoura National University, Gamasa, Egypt

**Keywords:** autophagy, dexamethasone, empagliflozin, ferroptosis, inflammation, non-alcoholic steatohepatitis

There was a mistake in Table 2 as published. Upon review, we identified an error in the reported primer sequences of the PCR markers in the Methods section as follows:

**TABLE 2:** Forward and reverse primers for PCR.

Primer	Forward	Reverse
FTH1	ATC​AAC​CGC​CAG​ATC​AAC​CT	TCT​CCC​AGT​CAT​CAC​GGT​CA
Calcineurin-A	AGA​TGG​ATT​TGA​CGG​AGC​CAC	GCT​GCT​ATT​ACT​GCC​GTT​GC
NCOA-4	AGT​GTC​TGG​GTC​GGT​CCA​A	GTG​AAT​CTG​AGC​TTT​CAC​CTC​TCG​T
FABP-1	CTG​GGG​AAA​AGG​TCA​AGG​CAG	TTG​TAG​ACG​ATG​TCA​CCC​AGT​G
GAPDH	TCT​TCT​TGT​GCA​GTG​CCA​GC	TGC​CGT​TGA​ACT​TGC​CGT​GG

The reverse primer of Calcineurin-A was reported as ATC​CTT​TGA​TGG​CGC​TTT​GC. The correct sequence is: GCT​GCT​ATT​ACT​GCC​GTT​GC.

The forward primer of NCOA-4 was reported as TGT​TCA​CGC​AGC​AGT​TTT​CGT​C. The correct sequence is: AGT​GTC​TGG​GTC​GGT​CCA​A.

Additionally, the forward primer sequence of FABP-1 was inadvertently duplicated and mistakenly inserted as the forward primer of GADPH. The correct sequence is: TCT​TCT​TGT​GCA​GTG​CCA​GC.

The corrected Table 2 appears below.

The original article has been updated.

